# 4-Hydr­oxy-3-(1′-methyl-2-oxo-4′-phenyl­spiro­[indoline-3,2′-pyrrolidine]-3′-yl­carbon­yl)quinolin-2(1*H*)-one

**DOI:** 10.1107/S1600536810010500

**Published:** 2010-03-27

**Authors:** K. Revathi, M. Sankaran, P. Ramesh, P. S. Mohan, M. N. Ponnuswamy

**Affiliations:** a Centre of Advanced Study in Crystallography and Biophysics, University of Madras, Guindy Campus, Chennai 600 025, India; b Department of Chemistry, School of Chemical Sciences, Bharathiar University, Coimbatore 641 046, India

## Abstract

In the title compound, C_28_H_23_N_3_O_4_, the dihedral angle between the quinoline and indole ring systems is 29.30 (5)°. The pyrrolidine ring adopts a twist conformation. An intra­molecular O—H⋯O hydrogen bond generates an *S*(6) ring motif. A weak intra­molecular C3—H3⋯O3 inter­action is also observed. In the crystal, mol­ecules are linked by two sets of N—H⋯O hydrogen bonds, forming centrosymmetric dimers containing two *R*_2_^2^(8) ring motifs. The dimers are linked *via* C—H⋯π inter­actions.

## Related literature

For general background to indole, quinoline and pyrrolidine derivatives, see: Amalraj *et al.* (2003 ▶); Cordell (1981 ▶); Suzuki *et al.* (1994 ▶). For puckering parameters, see: Cremer & Pople (1975 ▶). For asymmetry parameters, see: Nardelli (1983 ▶). For hydrogen-bond motifs, see: Bernstein *et al.* (1995 ▶).
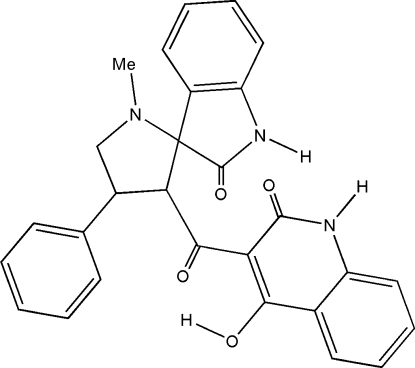


## Experimental

### 

#### Crystal data

C_28_H_23_N_3_O_4_
*M* *_r_* = 465.49
Triclinic, 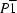

*a* = 9.6918 (3) Å
*b* = 11.0258 (3) Å
*c* = 12.9663 (4) Å
α = 69.111 (1)°
β = 72.044 (2)°
γ = 66.410 (1)°
*V* = 1163.93 (6) Å^3^
*Z* = 2
Mo *K*α radiation
μ = 0.09 mm^−1^
*T* = 293 K
0.20 × 0.20 × 0.20 mm

#### Data collection

Bruker SMART APEXII area-detector diffractometer
Absorption correction: multi-scan (*SADABS*; Bruker, 2008 ▶) *T*_min_ = 0.982, *T*_max_ = 0.982
21655 measured reflections
5795 independent reflections
4635 reflections with *I* > 2σ(*I*)
*R*_int_ = 0.023

#### Refinement

*R*[*F* ^2^ > 2σ(*F* ^2^)] = 0.040
*wR*(*F* ^2^) = 0.119
*S* = 1.03
5795 reflections
329 parameters
H atoms treated by a mixture of independent and constrained refinement
Δρ_max_ = 0.22 e Å^−3^
Δρ_min_ = −0.19 e Å^−3^

### 

Data collection: *APEX2* (Bruker, 2008 ▶); cell refinement: *SAINT* (Bruker, 2008 ▶); data reduction: *SAINT*; program(s) used to solve structure: *SHELXS97* (Sheldrick, 2008 ▶); program(s) used to refine structure: *SHELXL97* (Sheldrick, 2008 ▶); molecular graphics: *ORTEP-3* (Farrugia, 1997 ▶); software used to prepare material for publication: *SHELXL97* and *PLATON* (Spek, 2009 ▶).

## Supplementary Material

Crystal structure: contains datablocks global, I. DOI: 10.1107/S1600536810010500/ci5061sup1.cif

Structure factors: contains datablocks I. DOI: 10.1107/S1600536810010500/ci5061Isup2.hkl

Additional supplementary materials:  crystallographic information; 3D view; checkCIF report

## Figures and Tables

**Table 1:** Hydrogen-bond geometry (Å, °) *Cg*6 is the centroid of the C26–C31 ring.

*D*—H⋯*A*	*D*—H	H⋯*A*	*D*⋯*A*	*D*—H⋯*A*
O4—H4*A*⋯O2	0.99 (2)	1.56 (2)	2.4840 (14)	155 (2)
N8—H8⋯O3^i^	0.89 (2)	1.92 (2)	2.7837 (13)	165 (2)
N18—H18⋯O1^i^	0.90 (2)	1.95 (2)	2.8497 (14)	177 (2)
C3—H3⋯O3	0.98	2.21	2.7944 (13)	117
C21—H21⋯*Cg*6^ii^	0.93	2.72	3.5360 (18)	147

Symmetry codes: (i) 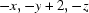; (ii) 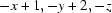.

**Fig. 1.**
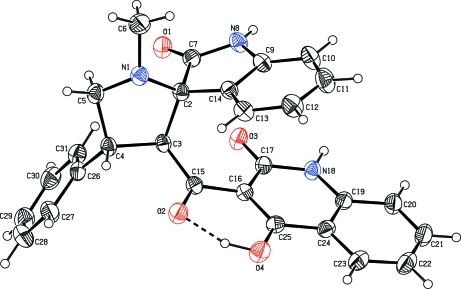
The molecular structure of the title compound, showing the atomic numbering and displacement ellipsoids drawn at the 50% probability level. The dashed line indicates a hydrogen bond.

**Fig. 2.**
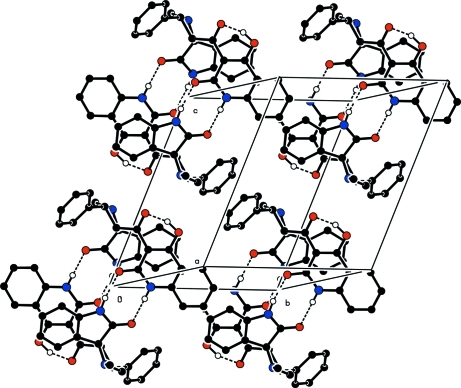
The crystal packing of the title compound. H atoms not involved in hydrogen bonding (dashed lines) have been omitted for clarity.

## supplementary crystallographic information

### Comment

Substituted pyrrolidine compounds possess antimicrobial and antifungal activities against various pathogens (Amalraj *et al.*, 2003). Several optically active pyrrolidine compounds are used as intermediates in controlled asymmetric synthesis (Suzuki *et al.*, 1994). The spiro- indole-pyrrolidine ring system is a frequently encountered structural motif in many biologically important and pharmacologically relevant alkaloids, e.g. vincrinstine, vinblastine and spirotypostatins (Cordell, 1981). Against this background and to ascertain the detailed information on its molecular conformation, the X-ray structure determination of the title compound has been carried out.

The pyrrolidine ring (N1/C2-C5) in the molecule adopts a twist conformation. The puckering parameters (Cremer & Pople, 1975) and the asymmetry parameters (Nardelli, 1983) for this ring are q_2_ = 0.426 (1) Å, φ = 335.5 (2)° and Δ_2_(C3) = 6.8 (2)°. The sum of the bond angles around atom N1 (337.3°) of the pyrrolidine ring indicates sp^3^ hybridization. The indole and quinoline ring systems are planar and keto atoms O1 and O3 deviate from the attached ring system by 0.011 (1) and -0.122 (1) Å, respectively. The dihedral angle between the indole and quinoline ring systems is 29.30 (5)°. An intramolecular O4—H4···O2 hydrogen bond generates an S(6) ring motif (Bernstein *et al.*, 1995). A weak intramolecular C3—H3···O3 interaction is also observed.

The molecules at (x, y, z) and (-x, 2-y, -z) are linked by two sets of N8—H8···O3 and N18—H18···O1 hydrogen bonds to form a centrosymmetric dimer containing two R_2_^2^(8) ring motifs (Fig. 2).

### Experimental

A mixture of 3-cinnamoyl-4-hydroxyquinolin-2(1*H*)-one (0.5 mmol), isatin (0.5 mmol) and sarcosine (0.55 mmol) was refluxed in methanol until the disappearance of the starting materials as evidenced by the TLC. After completion of the reaction, the solvent was removed in vacuo and the residue was chromatographed on silica gel using hexane-ethyl acetate mixture (7:3) as eluent to give the title compound. The compound was recrystallized in DMF-methanol (3:7 v/v).

### Refinement

N- and O-bound H atoms were located in a difference map and refined isotropically. C-bound H atoms were positioned geometrically (C–H = 0.93–0.98 Å) and allowed to ride on their parent atoms, with U_iso_(H) = 1.5U_eq_(C) for methyl H and 1.2U_eq_(C) for other H atoms.

### Crystal data

**Table:**

C_28_H_23_N_3_O_4_	*Z* = 2
*M**_r_* = 465.49	*F*(000) = 488
Triclinic, *P*1	*D*_x_ = 1.328 Mg m^−^^3^
Hall symbol: -P 1	Mo *K*α radiation, λ = 0.71073 Å
*a* = 9.6918 (3) Å	Cell parameters from 1865 reflections
*b* = 11.0258 (3) Å	θ = 1.7–28.4°
*c* = 12.9663 (4) Å	µ = 0.09 mm^−^^1^
α = 69.111 (1)°	*T* = 293 K
β = 72.044 (2)°	Block, colourless
γ = 66.410 (1)°	0.20 × 0.20 × 0.20 mm
*V* = 1163.93 (6) Å^3^	

### Data collection

**Table:**

Bruker SMART APEXII area-detector diffractometer	5795 independent reflections
Radiation source: fine-focus sealed tube	4635 reflections with *I* > 2σ(*I*)
graphite	*R*_int_ = 0.023
ω and φ scans	θ_max_ = 28.4°, θ_min_ = 1.7°
Absorption correction: multi-scan (*SADABS*; Bruker, 2008)	*h* = −12→12
*T*_min_ = 0.982, *T*_max_ = 0.982	*k* = −14→14
21655 measured reflections	*l* = −17→17

### Refinement

**Table:**

Refinement on *F*^2^	Primary atom site location: structure-invariant direct methods
Least-squares matrix: full	Secondary atom site location: difference Fourier map
*R*[*F*^2^ > 2σ(*F*^2^)] = 0.040	Hydrogen site location: inferred from neighbouring sites
*wR*(*F*^2^) = 0.119	H atoms treated by a mixture of independent and constrained refinement
*S* = 1.03	*w* = 1/[σ^2^(*F*_o_^2^) + (0.0596*P*)^2^ + 0.2023*P*] where *P* = (*F*_o_^2^ + 2*F*_c_^2^)/3
5795 reflections	(Δ/σ)_max_ = 0.008
329 parameters	Δρ_max_ = 0.22 e Å^−^^3^
0 restraints	Δρ_min_ = −0.19 e Å^−^^3^

### Special details

**Table:**

Geometry. All esds (except the esd in the dihedral angle between two l.s. planes) are estimated using the full covariance matrix. The cell esds are taken into account individually in the estimation of esds in distances, angles and torsion angles; correlations between esds in cell parameters are only used when they are defined by crystal symmetry. An approximate (isotropic) treatment of cell esds is used for estimating esds involving l.s. planes.
Refinement. Refinement of *F*^2^ against ALL reflections. The weighted *R*-factor wR and goodness of fit *S* are based on *F*^2^, conventional *R*-factors *R* are based on *F*, with *F* set to zero for negative *F*^2^. The threshold expression of *F*^2^ > σ(*F*^2^) is used only for calculating *R*-factors(gt) etc. and is not relevant to the choice of reflections for refinement. *R*-factors based on *F*^2^ are statistically about twice as large as those based on *F*, and *R*- factors based on ALL data will be even larger.

### Fractional atomic coordinates and isotropic or equivalent isotropic displacement parameters (Å^2^)

**Table:**

	*x*	*y*	*z*	*U*_iso_*/*U*_eq_	
O1	−0.12393 (11)	0.84110 (9)	0.20477 (8)	0.0513 (2)	
O2	0.31591 (12)	0.88012 (10)	0.35337 (8)	0.0540 (2)	
O3	0.17428 (11)	0.93755 (10)	0.06203 (7)	0.0489 (2)	
O4	0.43677 (12)	1.06028 (12)	0.24273 (9)	0.0577 (3)	
N1	−0.11028 (12)	0.89412 (11)	0.41897 (8)	0.0438 (2)	
C2	−0.03598 (13)	0.94481 (12)	0.30309 (9)	0.0377 (2)	
C3	0.13212 (13)	0.83758 (11)	0.29618 (9)	0.0362 (2)	
H3	0.1534	0.7989	0.2334	0.043*	
C4	0.12577 (14)	0.72173 (12)	0.40670 (9)	0.0398 (3)	
H4	0.1635	0.7380	0.4609	0.048*	
C5	−0.04598 (15)	0.74471 (13)	0.44758 (10)	0.0451 (3)	
H5A	−0.0828	0.7067	0.4086	0.054*	
H5B	−0.0702	0.7047	0.5280	0.054*	
C6	−0.27726 (17)	0.95032 (17)	0.44161 (13)	0.0608 (4)	
H6A	−0.3175	0.9223	0.3981	0.091*	
H6B	−0.3085	1.0488	0.4214	0.091*	
H6C	−0.3159	0.9170	0.5202	0.091*	
C7	−0.10803 (14)	0.94192 (12)	0.21255 (9)	0.0405 (3)	
N8	−0.15228 (13)	1.07050 (11)	0.14433 (9)	0.0465 (3)	
C9	−0.11390 (14)	1.16162 (12)	0.17404 (10)	0.0437 (3)	
C10	−0.13151 (19)	1.29824 (14)	0.12047 (13)	0.0604 (4)	
H10	−0.1705	1.3416	0.0546	0.072*	
C11	−0.0888 (2)	1.36838 (15)	0.16859 (15)	0.0669 (4)	
H11	−0.0970	1.4600	0.1332	0.080*	
C12	−0.03449 (19)	1.30537 (14)	0.26777 (14)	0.0594 (4)	
H12	−0.0103	1.3559	0.2997	0.071*	
C13	−0.01539 (15)	1.16707 (13)	0.32065 (11)	0.0480 (3)	
H13	0.0216	1.1243	0.3874	0.058*	
C14	−0.05276 (14)	1.09499 (12)	0.27148 (9)	0.0395 (2)	
C15	0.25320 (13)	0.90191 (11)	0.27531 (9)	0.0378 (2)	
C16	0.29296 (13)	0.99495 (11)	0.16590 (9)	0.0363 (2)	
C17	0.24039 (13)	1.01026 (11)	0.06679 (9)	0.0377 (2)	
N18	0.26902 (12)	1.11132 (10)	−0.02685 (8)	0.0419 (2)	
C19	0.34942 (13)	1.19400 (12)	−0.03571 (10)	0.0408 (3)	
C20	0.37500 (16)	1.29242 (13)	−0.13735 (12)	0.0515 (3)	
H20	0.3361	1.3035	−0.1986	0.062*	
C21	0.45814 (18)	1.37192 (15)	−0.14509 (13)	0.0597 (4)	
H21	0.4756	1.4372	−0.2122	0.072*	
C22	0.51668 (19)	1.35655 (16)	−0.05436 (14)	0.0624 (4)	
H22	0.5725	1.4117	−0.0613	0.075*	
C23	0.49284 (16)	1.26065 (15)	0.04533 (13)	0.0533 (3)	
H23	0.5326	1.2506	0.1058	0.064*	
C24	0.40805 (13)	1.17747 (12)	0.05590 (10)	0.0412 (3)	
C25	0.37906 (13)	1.07426 (12)	0.15761 (10)	0.0403 (3)	
C26	0.22252 (15)	0.58092 (12)	0.38987 (10)	0.0435 (3)	
C27	0.33649 (18)	0.49550 (14)	0.45126 (13)	0.0590 (4)	
H27	0.3503	0.5229	0.5064	0.071*	
C28	0.4310 (2)	0.36850 (16)	0.43128 (17)	0.0739 (5)	
H28	0.5078	0.3124	0.4726	0.089*	
C29	0.4112 (2)	0.32639 (15)	0.35130 (16)	0.0695 (4)	
H29	0.4755	0.2426	0.3373	0.083*	
C30	0.29633 (18)	0.40778 (15)	0.29184 (14)	0.0594 (4)	
H30	0.2814	0.3783	0.2385	0.071*	
C31	0.20241 (16)	0.53399 (13)	0.31095 (11)	0.0492 (3)	
H31	0.1245	0.5883	0.2703	0.059*	
H4A	0.405 (3)	0.985 (2)	0.301 (2)	0.106 (7)*	
H8	−0.1719 (19)	1.0831 (17)	0.0782 (15)	0.065 (5)*	
H18	0.2265 (18)	1.1249 (15)	−0.0844 (14)	0.054 (4)*	

### Atomic displacement parameters (Å^2^)

**Table:**

	*U*^11^	*U*^22^	*U*^33^	*U*^12^	*U*^13^	*U*^23^
O1	0.0677 (6)	0.0516 (5)	0.0457 (5)	−0.0282 (4)	−0.0212 (4)	−0.0070 (4)
O2	0.0656 (6)	0.0651 (6)	0.0408 (5)	−0.0325 (5)	−0.0224 (4)	−0.0019 (4)
O3	0.0688 (6)	0.0569 (5)	0.0340 (4)	−0.0363 (5)	−0.0133 (4)	−0.0061 (4)
O4	0.0679 (6)	0.0729 (7)	0.0496 (5)	−0.0398 (5)	−0.0200 (5)	−0.0094 (5)
N1	0.0470 (6)	0.0469 (6)	0.0327 (5)	−0.0145 (4)	−0.0058 (4)	−0.0078 (4)
C2	0.0430 (6)	0.0406 (6)	0.0306 (5)	−0.0140 (5)	−0.0105 (4)	−0.0075 (4)
C3	0.0437 (6)	0.0344 (5)	0.0303 (5)	−0.0128 (4)	−0.0101 (4)	−0.0060 (4)
C4	0.0495 (6)	0.0391 (6)	0.0311 (5)	−0.0170 (5)	−0.0125 (4)	−0.0029 (4)
C5	0.0527 (7)	0.0478 (6)	0.0328 (5)	−0.0211 (5)	−0.0077 (5)	−0.0036 (5)
C6	0.0495 (8)	0.0672 (9)	0.0528 (8)	−0.0153 (7)	−0.0008 (6)	−0.0134 (7)
C7	0.0427 (6)	0.0456 (6)	0.0346 (5)	−0.0155 (5)	−0.0099 (4)	−0.0088 (5)
N8	0.0553 (6)	0.0470 (6)	0.0392 (5)	−0.0141 (5)	−0.0214 (5)	−0.0065 (4)
C9	0.0465 (6)	0.0416 (6)	0.0411 (6)	−0.0093 (5)	−0.0138 (5)	−0.0102 (5)
C10	0.0755 (10)	0.0439 (7)	0.0574 (8)	−0.0109 (7)	−0.0311 (7)	−0.0028 (6)
C11	0.0842 (11)	0.0372 (7)	0.0783 (11)	−0.0144 (7)	−0.0298 (9)	−0.0087 (7)
C12	0.0701 (9)	0.0455 (7)	0.0703 (9)	−0.0146 (6)	−0.0219 (7)	−0.0222 (7)
C13	0.0535 (7)	0.0459 (7)	0.0469 (7)	−0.0106 (5)	−0.0150 (5)	−0.0169 (5)
C14	0.0420 (6)	0.0387 (6)	0.0355 (5)	−0.0098 (4)	−0.0089 (4)	−0.0100 (4)
C15	0.0423 (6)	0.0371 (5)	0.0345 (5)	−0.0112 (4)	−0.0100 (4)	−0.0097 (4)
C16	0.0380 (6)	0.0364 (5)	0.0350 (5)	−0.0115 (4)	−0.0076 (4)	−0.0102 (4)
C17	0.0409 (6)	0.0386 (6)	0.0331 (5)	−0.0138 (4)	−0.0059 (4)	−0.0093 (4)
N18	0.0495 (6)	0.0438 (5)	0.0344 (5)	−0.0199 (4)	−0.0101 (4)	−0.0058 (4)
C19	0.0386 (6)	0.0362 (5)	0.0431 (6)	−0.0114 (4)	−0.0028 (5)	−0.0110 (5)
C20	0.0535 (8)	0.0456 (7)	0.0473 (7)	−0.0178 (6)	−0.0058 (6)	−0.0046 (5)
C21	0.0603 (8)	0.0479 (7)	0.0601 (8)	−0.0249 (6)	0.0001 (7)	−0.0038 (6)
C22	0.0618 (9)	0.0573 (8)	0.0727 (10)	−0.0348 (7)	−0.0006 (7)	−0.0157 (7)
C23	0.0503 (7)	0.0564 (8)	0.0598 (8)	−0.0265 (6)	−0.0044 (6)	−0.0179 (6)
C24	0.0374 (6)	0.0399 (6)	0.0462 (6)	−0.0137 (5)	−0.0034 (5)	−0.0142 (5)
C25	0.0389 (6)	0.0436 (6)	0.0408 (6)	−0.0134 (5)	−0.0077 (5)	−0.0142 (5)
C26	0.0488 (7)	0.0377 (6)	0.0413 (6)	−0.0192 (5)	−0.0094 (5)	−0.0012 (5)
C27	0.0668 (9)	0.0463 (7)	0.0622 (9)	−0.0177 (6)	−0.0276 (7)	−0.0006 (6)
C28	0.0695 (10)	0.0471 (8)	0.0905 (12)	−0.0093 (7)	−0.0319 (9)	0.0010 (8)
C29	0.0670 (10)	0.0402 (7)	0.0916 (12)	−0.0172 (7)	−0.0074 (9)	−0.0137 (7)
C30	0.0652 (9)	0.0512 (8)	0.0671 (9)	−0.0294 (7)	−0.0005 (7)	−0.0204 (7)
C31	0.0530 (7)	0.0451 (7)	0.0511 (7)	−0.0209 (6)	−0.0090 (6)	−0.0102 (5)

### Geometric parameters (Å, °)

**Table:**

O1—C7	1.2232 (15)	C12—H12	0.93
O2—C15	1.2412 (14)	C13—C14	1.3797 (17)
O3—C17	1.2360 (14)	C13—H13	0.93
O4—C25	1.3210 (15)	C15—C16	1.4679 (15)
O4—H4A	0.99 (2)	C16—C25	1.3908 (16)
N1—C6	1.4572 (18)	C16—C17	1.4559 (15)
N1—C5	1.4577 (16)	C17—N18	1.3666 (15)
N1—C2	1.4645 (15)	N18—C19	1.3748 (16)
C2—C14	1.5092 (16)	N18—H18	0.900 (16)
C2—C7	1.5546 (16)	C19—C24	1.3950 (17)
C2—C3	1.5824 (16)	C19—C20	1.4035 (17)
C3—C15	1.5123 (16)	C20—C21	1.371 (2)
C3—C4	1.5466 (15)	C20—H20	0.93
C3—H3	0.98	C21—C22	1.388 (2)
C4—C26	1.5124 (17)	C21—H21	0.93
C4—C5	1.5265 (18)	C22—C23	1.371 (2)
C4—H4	0.98	C22—H22	0.93
C5—H5A	0.97	C23—C24	1.4068 (18)
C5—H5B	0.97	C23—H23	0.93
C6—H6A	0.96	C24—C25	1.4389 (17)
C6—H6B	0.96	C26—C27	1.3840 (18)
C6—H6C	0.96	C26—C31	1.3907 (18)
C7—N8	1.3526 (16)	C27—C28	1.397 (2)
N8—C9	1.3999 (17)	C27—H27	0.93
N8—H8	0.886 (18)	C28—C29	1.366 (3)
C9—C10	1.3791 (18)	C28—H28	0.93
C9—C14	1.3922 (16)	C29—C30	1.369 (2)
C10—C11	1.385 (2)	C29—H29	0.93
C10—H10	0.93	C30—C31	1.385 (2)
C11—C12	1.379 (2)	C30—H30	0.93
C11—H11	0.93	C31—H31	0.93
C12—C13	1.3915 (19)		
			
C25—O4—H4A	102.8 (13)	C14—C13—H13	120.9
C6—N1—C5	115.61 (11)	C12—C13—H13	120.9
C6—N1—C2	115.01 (10)	C13—C14—C9	120.22 (11)
C5—N1—C2	106.72 (9)	C13—C14—C2	131.02 (10)
N1—C2—C14	113.47 (9)	C9—C14—C2	108.75 (10)
N1—C2—C7	113.94 (10)	O2—C15—C16	119.22 (11)
C14—C2—C7	101.31 (9)	O2—C15—C3	119.46 (10)
N1—C2—C3	102.76 (8)	C16—C15—C3	121.25 (9)
C14—C2—C3	117.72 (10)	C25—C16—C17	119.02 (10)
C7—C2—C3	107.99 (9)	C25—C16—C15	118.98 (10)
C15—C3—C4	114.61 (9)	C17—C16—C15	121.97 (10)
C15—C3—C2	112.81 (9)	O3—C17—N18	118.80 (10)
C4—C3—C2	105.06 (9)	O3—C17—C16	124.44 (10)
C15—C3—H3	108.0	N18—C17—C16	116.76 (10)
C4—C3—H3	108.0	N18—C19—C24	119.67 (11)
C2—C3—H3	108.0	N18—C19—C20	119.99 (12)
C26—C4—C5	115.35 (10)	C24—C19—C20	120.32 (12)
C26—C4—C3	112.53 (9)	C21—C20—C19	119.04 (14)
C5—C4—C3	102.87 (9)	C21—C20—H20	120.5
C26—C4—H4	108.6	C19—C20—H20	120.5
C5—C4—H4	108.6	C20—C21—C22	121.10 (13)
C3—C4—H4	108.6	C20—C21—H21	119.4
N1—C5—C4	102.03 (10)	C22—C21—H21	119.4
N1—C5—H5A	111.4	C23—C22—C21	120.47 (14)
C4—C5—H5A	111.4	C23—C22—H22	119.8
N1—C5—H5B	111.4	C21—C22—H22	119.8
C4—C5—H5B	111.4	C22—C23—C24	119.78 (14)
H5A—C5—H5B	109.2	C22—C23—H23	120.1
N1—C6—H6A	109.5	C24—C23—H23	120.1
N1—C6—H6B	109.5	C19—C24—C23	119.28 (12)
H6A—C6—H6B	109.5	C19—C24—C25	117.81 (11)
N1—C6—H6C	109.5	C23—C24—C25	122.91 (12)
H6A—C6—H6C	109.5	O4—C25—C16	121.98 (11)
H6B—C6—H6C	109.5	O4—C25—C24	116.49 (11)
O1—C7—N8	125.56 (11)	C16—C25—C24	121.52 (11)
O1—C7—C2	126.01 (10)	C27—C26—C31	117.78 (13)
N8—C7—C2	108.42 (10)	C27—C26—C4	120.84 (12)
C7—N8—C9	111.31 (10)	C31—C26—C4	121.36 (11)
C7—N8—H8	119.7 (11)	C26—C27—C28	120.68 (15)
C9—N8—H8	125.8 (11)	C26—C27—H27	119.7
C10—C9—C14	121.77 (12)	C28—C27—H27	119.7
C10—C9—N8	128.28 (12)	C29—C28—C27	120.29 (15)
C14—C9—N8	109.94 (10)	C29—C28—H28	119.9
C9—C10—C11	117.42 (13)	C27—C28—H28	119.9
C9—C10—H10	121.3	C28—C29—C30	119.90 (15)
C11—C10—H10	121.3	C28—C29—H29	120.0
C12—C11—C10	121.45 (14)	C30—C29—H29	120.0
C12—C11—H11	119.3	C29—C30—C31	120.13 (15)
C10—C11—H11	119.3	C29—C30—H30	119.9
C11—C12—C13	120.76 (13)	C31—C30—H30	119.9
C11—C12—H12	119.6	C30—C31—C26	121.18 (13)
C13—C12—H12	119.6	C30—C31—H31	119.4
C14—C13—C12	118.27 (12)	C26—C31—H31	119.4
			
C6—N1—C2—C14	67.51 (14)	C4—C3—C15—O2	−14.08 (15)
C5—N1—C2—C14	−162.83 (10)	C2—C3—C15—O2	106.11 (12)
C6—N1—C2—C7	−47.74 (15)	C4—C3—C15—C16	168.83 (10)
C5—N1—C2—C7	81.91 (12)	C2—C3—C15—C16	−70.98 (13)
C6—N1—C2—C3	−164.29 (11)	O2—C15—C16—C25	−10.92 (17)
C5—N1—C2—C3	−34.63 (11)	C3—C15—C16—C25	166.18 (10)
N1—C2—C3—C15	−116.48 (10)	O2—C15—C16—C17	171.31 (11)
C14—C2—C3—C15	9.00 (13)	C3—C15—C16—C17	−11.59 (16)
C7—C2—C3—C15	122.80 (10)	C25—C16—C17—O3	173.82 (11)
N1—C2—C3—C4	9.05 (11)	C15—C16—C17—O3	−8.41 (18)
C14—C2—C3—C4	134.53 (10)	C25—C16—C17—N18	−5.28 (16)
C7—C2—C3—C4	−111.68 (10)	C15—C16—C17—N18	172.49 (10)
C15—C3—C4—C26	−93.05 (12)	O3—C17—N18—C19	−175.84 (11)
C2—C3—C4—C26	142.55 (10)	C16—C17—N18—C19	3.31 (17)
C15—C3—C4—C5	142.18 (10)	C17—N18—C19—C24	−0.20 (18)
C2—C3—C4—C5	17.79 (11)	C17—N18—C19—C20	178.42 (11)
C6—N1—C5—C4	176.30 (10)	N18—C19—C20—C21	−178.69 (12)
C2—N1—C5—C4	46.99 (11)	C24—C19—C20—C21	−0.08 (19)
C26—C4—C5—N1	−161.42 (9)	C19—C20—C21—C22	−0.1 (2)
C3—C4—C5—N1	−38.53 (11)	C20—C21—C22—C23	0.2 (2)
N1—C2—C7—O1	−56.11 (16)	C21—C22—C23—C24	−0.2 (2)
C14—C2—C7—O1	−178.34 (12)	N18—C19—C24—C23	178.72 (11)
C3—C2—C7—O1	57.35 (15)	C20—C19—C24—C23	0.10 (18)
N1—C2—C7—N8	123.09 (11)	N18—C19—C24—C25	−0.94 (16)
C14—C2—C7—N8	0.87 (12)	C20—C19—C24—C25	−179.56 (11)
C3—C2—C7—N8	−123.44 (11)	C22—C23—C24—C19	0.0 (2)
O1—C7—N8—C9	−178.42 (12)	C22—C23—C24—C25	179.67 (13)
C2—C7—N8—C9	2.37 (14)	C17—C16—C25—O4	−176.49 (11)
C7—N8—C9—C10	175.61 (14)	C15—C16—C25—O4	5.67 (17)
C7—N8—C9—C14	−5.01 (15)	C17—C16—C25—C24	4.40 (17)
C14—C9—C10—C11	−1.3 (2)	C15—C16—C25—C24	−173.44 (10)
N8—C9—C10—C11	178.02 (15)	C19—C24—C25—O4	179.58 (11)
C9—C10—C11—C12	−1.6 (3)	C23—C24—C25—O4	−0.07 (18)
C10—C11—C12—C13	2.4 (3)	C19—C24—C25—C16	−1.26 (17)
C11—C12—C13—C14	−0.2 (2)	C23—C24—C25—C16	179.10 (12)
C12—C13—C14—C9	−2.6 (2)	C5—C4—C26—C27	−118.66 (13)
C12—C13—C14—C2	175.60 (13)	C3—C4—C26—C27	123.74 (13)
C10—C9—C14—C13	3.5 (2)	C5—C4—C26—C31	62.68 (15)
N8—C9—C14—C13	−175.95 (11)	C3—C4—C26—C31	−54.92 (15)
C10—C9—C14—C2	−175.11 (13)	C31—C26—C27—C28	2.1 (2)
N8—C9—C14—C2	5.46 (14)	C4—C26—C27—C28	−176.58 (14)
N1—C2—C14—C13	55.29 (17)	C26—C27—C28—C29	−0.6 (3)
C7—C2—C14—C13	177.84 (13)	C27—C28—C29—C30	−1.2 (3)
C3—C2—C14—C13	−64.72 (17)	C28—C29—C30—C31	1.4 (2)
N1—C2—C14—C9	−126.32 (11)	C29—C30—C31—C26	0.2 (2)
C7—C2—C14—C9	−3.78 (12)	C27—C26—C31—C30	−2.0 (2)
C3—C2—C14—C9	113.66 (11)	C4—C26—C31—C30	176.74 (12)

### Hydrogen-bond geometry (Å, °)

**Table:**

Cg6 is the centroid of the C26–C31 ring.

**Table:**

*D*—H···*A*	*D*—H	H···*A*	*D*···*A*	*D*—H···*A*
O4—H4A···O2	0.99 (2)	1.56 (2)	2.4840 (14)	155 (2)
N8—H8···O3^i^	0.89 (2)	1.92 (2)	2.7837 (13)	165 (2)
N18—H18···O1^i^	0.90 (2)	1.95 (2)	2.8497 (14)	177 (2)
C3—H3···O3	0.98	2.21	2.7944 (13)	117
C21—H21···Cg6^ii^	0.93	2.72	3.5360 (18)	147

Symmetry codes:  (i) −*x*, −*y*+2, −*z*; (ii) −*x*+1, −*y*+2, −*z*.
